# From Clinical Trials to Clinical Practice–Patterns of Practice Survey on Prostate SABR in Australia and New Zealand

**DOI:** 10.1016/j.adro.2026.102144

**Published:** 2026-07-17

**Authors:** Therese Kang, Shreya Armstrong, Sarat Chander, Jeremy de Leon, Renee Finnigan, Suki Gill, Mario Guerrieri, Amy Hayden, Braden Higgs, Tanya Holt, Michael Jones, Yuan Hong Lin, Giuseppe Sasso, Neetu Tejani, Vedang Murthy, Andrew Loblaw, Wee Loon Ong

**Affiliations:** a Alfred Health Radiation Oncology, School of Translational Medicine, Monash University, Melbourne, Victoria, Australia; b Gippsland Radiation Oncology, Latrobe Regional Health, Traralgon, Victoria, Australia; c Department of Radiation Oncology, Andrew Love Cancer Centre, Barwon Health, Geelong, Victoria, Australia; d School of Public Health and Preventive Medicine, Monash University, Melbourne, Victoria, Australia; e Department of Radiation Oncology, North Coast Cancer Institute, Lismore Base Hospital, Lismore, New South Wales, Australia; f Division of Radiation Oncology, Peter MacCallum Cancer Centre, Melbourne, Victoria, Australia; g Sir Peter MacCallum Department of Oncology, University of Melbourne, Melbourne, Victoria, Australia; h GenesisCare, Darlinghurst, New South Wales, Australia; i ICON Gold Coast University Hospital, Southport, Queensland, Australia; j Department of Radiation Oncology, Sir Charles Gairdner Hospital, Nedlands, Western Australia, Australia; k University of Western Australia, Crawley, Western Australia, Australia; l GenesisCare, Western Private Hospital, Footscray, Victoria, Australia; m Sydney West Radiation Oncology Network, Western Sydney Local Health District, New South Wales, Australia; n Western Sydney University, Sydney, New South Wales, Australia; o Department of Radiation Oncology, Royal Adelaide Hospital, Adelaide, South Australia, Australia; p University of South Australia, Adelaide, South Australia, Australia; q Radiation Oncology, Princess Alexandra Hospital, Brisbane, Queensland, Australia; r The University of Queensland, Brisbane, Queensland, Australia; s Department of Radiation Oncology, Royal Hobart Hospital, Hobart, Tasmania, Australia; t University of Tasmania, Hobart, Tasmania, Australia; u Department of Oncology, School of Medical and Health Sciences, The University of Auckland, Auckland, New Zealand; v Auckland Radiation Oncology, Auckland, New Zealand; w Radiation Oncology Services, Auckland and Dunedin Hospitals, Health New Zealand–Te Whatu Ora, New Zealand; x Department of Radiation Oncology, Advanced Centre for Treatment Research and Education in Cancer (ACTREC), Homi Bhabha National Institute, Tata Memorial Hospital, Mumbai, India; y Department of Radiation Oncology, Sunnybrook Odette Cancer Centre, University of Toronto, Toronto, Ontario, Canada; z Institute of Health Policy, Management and Evaluation, University of Toronto, Toronto, Ontario, Canada

## Abstract

**Purpose:**

Prostate stereotactic ablative body radiotherapy (SABR) is now a standard-of-care radiation therapy option for prostate cancer based on high-level clinical trial evidence. As part of the Australia and New Zealand (ANZ) prostate SABR guideline development process, the Royal Australian and New Zealand College of Radiologists has commissioned a pattern-of-practice survey on contemporaneous prostate SABR practice.

**Methods and Materials:**

We conducted a cross-sectional survey among ANZ genitourinary radiation oncologists (ROs), focusing on patient selection, contouring, treatment planning, and delivery of prostate SABR.

**Results:**

A total of 53 ROs across ANZ responded to the survey (response rate: 28%). Of the 41 ROs currently offering prostate SABR, 95%, 90%, 35%, 5%, and 0% offer prostate SABR as standard-of-care options to individuals with favorable intermediate risk, unfavorable intermediate risk, favorable high risk (NINJA/ TROG18.01 trial eligible), very high risk, and node-positive prostate cancer, respectively. Most ROs (86%) routinely use fiducial markers. All ROs routinely request planning magnetic resonance imaging for contouring. Clinical target volume contouring ranged from prostate alone (26%) to prostate plus proximal 1 cm of the seminal vesicles (81%). Planning target volume margins ranged from 3 to 5 mm. An anisotropic 5 mm margin with 3 mm posteriorly was most common (56%). Nineteen (45%) ROs provide a focal boost to the intraprostatic lesion. The most common dose fractionation was 36.25 Gy to the planning target volume and 40 Gy to the clinical target volume over 5 fractions, delivered as 2 (50%) to 3 fractions (76%) per week. The majority of ROs offer prostate SABR on standard computed tomography-linear accelerator (LINAC) (88%), with several ROs also treating patients with CyberKnife (10%) and magnetic resonance-LINAC (5%). Of the ROs who use standard computed tomography-LINAC, 84% use intrafraction motion monitoring. Twelve (23%) ROs do not offer prostate SABR. The main barrier cited was a lack of expertise (42%).

**Conclusions:**

This ANZ-wide prostate SABR pattern-of-practice survey demonstrated some variations in practice, providing contemporary insight into how prostate SABR is delivered across ANZ and highlighting barriers to wider adoption.

## Introduction

Ultrahypofractionated stereotactic ablative body radiotherapy (SABR) is now an established standard-of-care treatment option for localized prostate cancer (PCa) supported by phase 3 randomized trial evidence.^1^ However, there is often a lag in adopting evidence in routine clinical practice. The implementation of prostate SABR can be complex, requiring specialized technology (eg, intrafraction motion management) and staff upskilling. A clinical practice guideline may expedite and facilitate the safe adoption of prostate SABR in routine clinical practice. In the lead-up to the development of the Royal Australian and New Zealand College of Radiologists Faculty of Radiation Oncology Genitourinary Group (FROGG) binational prostate SABR consensus clinical practice guidelines, the FROGG Executive Committee commissioned an Australia and New Zealand (ANZ)-wide survey to better understand the contemporaneous patterns of practice of prostate SABR in ANZ and the potential barriers to the adoption of prostate SABR.

## Methods and Materials

We conducted a cross-sectional survey of prostate SABR among practicing radiation oncologists (ROs) with a subspecialty interest in genitourinary cancers in ANZ. The survey consisted of prostate SABR-related questions, including patient selection, contouring, treatment planning, and delivery. The survey was developed by 2 ROs (TK and WO), and piloted and reviewed by the FROGG Executive Committee. The survey was then distributed to the FROGG membership mailing list on the SurveyMonkey platform between July 7, 2025, and August 10, 2025. To maximize the response rate, an email reminder was sent 2 weeks after the initial invitation. In addition, 1 week prior to survey closure, the FROGG Executive Committee contacted ROs from institutions that had not responded to obtain representative responses from as many institutions as possible across all ANZ jurisdictions. Deidentified survey responses were collated, and descriptive statistical analysis was performed using Stata/SE v18.5 (StataCorp LLC).

## Results

A total of 53 ROs across ANZ responded. The overall response rate was 28% (53/190) across 31 institutions in all ANZ jurisdictions, including metropolitan, regional, and public and private centers. Of the responders, 77% (41/53) ROs are currently offering prostate SABR as a standard-of-care option. Twelve ROs also offer prostate SABR in clinical trials, including the NINJA/ TROG18.01 trial (ACTRN12618001806257),^2^ SABRE trial (NCT04905069), and several single-institution phase 2 trials.

### Patient selection

Of the 41 ROs who offer prostate SABR, 40 (95%), 38 (90%), 15 (35%), 2 (5%), and 0 (0%) offer SABR as a standard-of-care option to individuals with favorable intermediate risk, unfavorable intermediate risk, favorable high risk, unfavorable high risk (outside of NINJA/ TROG18.01 trial^2^ eligibility criteria), and node-positive PCa, respectively (Table 1). Two-thirds of ROs (28/41) apply a prostate volume cutoff, with no ROs offering prostate SABR to patients with a prostate volume >100 cm^3^. Almost all ROs (40/41) assess baseline urinary function using the International Prostate Symptom Score (IPSS); however, approximately half (18/40) do not have a specific IPSS cutoff for prostate SABR. Twenty-seven (66%) ROs offer prostate SABR in the setting of hip replacement. Twenty-four (57%) ROs offer prostate SABR after previous transurethral resection of the prostate, with almost all waiting a minimum of 3 months between transurethral resection of the prostate and prostate SABR.

**Table 1:** Patient selection criteria (n = 41)

Patient selection criteria	n (%)
NCCN risk categories	
Favorable intermediate risk	40 (98)
Unfavorable intermediate risk	38 (93)
Favorable high risk (ie, NINJA trial eligible)	15 (37)
Unfavorable high risk (ie, NINJA trial ineligible)	2 (5)
Node positive	0 (0)
IPSS assessed for patient selection	
No	1 (2)
Yes	40 (98)
No IPSS cutoff	18 (45)
≤7	2 (5)
10-14	9 (23)
15-19	6 (15)
≤20	5 (13)
Do you have a prostate volume cutoff?	
No	13 (32)
Yes	28 (68)
<50 cm^3^	3 (11)
<60 cm^3^	4 (14)
<80 cm^3^	5 (18)
<90 cm^3^	2 (7)
<100 cm^3^	14 (50)
Do you offer prostate SABR in patients with hip replacement?	
No	14 (34)
Yes, unilateral hip replacement	17 (42)
Yes, bilateral hip replacement	10 (24)
Do you offer prostate SABR in patients who had previous TURP	
No	17 (41)
Yes	24 (59)
Duration between TURP and prostate SABR (n = 24)	
≥8 wk	1 (4)
≥3 mo	15 (63)
≥4 mo	2 (8)
≥6 mo	5 (21)
≥12 mo	1 (4)

*Abbreviations:* IPSS = International Prostate Symptom Score; TURP = transurethral resection of the prostate; NCCN = National Comprehensive Cancer Network.

*IPSS score: mild (0-7); moderate (8-19); severe (20-35).

### Planning and simulation

Routine fiducial marker use was reported by 86%of ROs (35/41); 3/41 (7%) ROs do not use fiducial markers, and 3/41 (7%) omit fiducial markers if patients are unable to pay (Table 2). Half of the ROs (22/41) routinely use rectal spacers, while one-third (13/41) offer them selectively based on patient request or health insurance coverage. The cost of rectal spacers is covered by private health insurance (97%, 34/35), hospital/departmental funds (31%, 11/35), or patients (51%, 18/35). All ROs routinely use planning magnetic resonance imaging (MRI); one RO would offer prostate SABR without planning MRI if there is a contraindication to MRI. The cost of planning MRI is covered by the treating department (60%, 24/40) or patients (68%, 27/40), while 4 ROs (10%, 4/40) have access to in-house MR simulators.

**Table 2:** Planning and simulation (n=41)

Planning and simulation	N (%)
The use fiducial marker	
No	3 (7%)
Yes, always	35 (86%)
Yes, sometimes	3 (7%)
The use of rectal spacer	
No	6 (15%)
Yes, always	22 (54%)
Yes, Sometimes	13 (32%)
Cost of rectal spacer covered by: (n=35)	
Private health insurance	34 (97%)
Patient out-of-pocket fee	18 (51%)
Hospital/ departmental fund	11 (31%)
The use of planning prostate MRI	
No	1 (2%)
Yes	40 (98%)
Cost of planning prostate MRI covered by: (n=40)	
In-house MR simulator	4 (10%)
Patient out-of-pocket fee	27 (68%)
Hospital/ departmental fund	24 (60%)

*Abbreviations:* MR = magnetic resonance; MRI = magnetic resonance imaging.

### Contouring and dose prescription

Clinical target volume contouring varied based on National Comprehensive Cancer Network (NCCN) risk categories, with the majority including the prostate and proximal 1 cm of the seminal vesicles (81%, 34/41) (Table 3). Planning target volume (PTV) expansion ranged from 3 to 5 mm; an anisotropic 5 mm expansion with 3 mm posterior expansion (56%, 23/41) is most commonly used. Nineteen (45%) ROs offer boosts to intraprostatic lesions as gross target volume (GTVboost), contoured using MRI and prostate-specific membrane antigen positron emission tomography (PSMA-PET) scans. In the event of discordance between MRI and PSMA-PET, half of the ROs (10/19) boolean the MRI and PSMA-PET GTVboost. For the intraprostatic GTVboost, most ROs (16/19) do not apply a PTV margin. All ROs contour the urethra as an organ-at-risk (OAR), predominantly using MRI, while 10% (4/41) use a computed tomography (CT) urethrogram at the time of planning CT.^3^ The most common dose fractionation is 40 Gy to the clinical target volume and 36.25 Gy to the PTV in 5 fractions (Table 4). The majority of ROs offer 2 (51%, 21/41) or 3 (78%, 32/41) fractions per week. The most common GTVboost doses are 42 Gy (26%, 5/19) and 45 Gy (42%, 8/19), respecting OAR dose constraints. One RO (2%) offers 42.7 Gy in 7 fractions, with a GTVboost up to 49 Gy. Most ROs apply their own departmental dose constraints, adapted from a combination of the Prostate Advances in Comparative Evidence (PACE-B)^1^ (48%, 20/41) and NINJA/ TROG18.01^2^ (36%, 15/41) trials.

**Table 3:** Target volume and organ-at-risk contouring (n=41)

Target volume and organ-at-risk contouring	N (%)
Clinical Target volume (CTV)	
Prostate alone	11 (26%)
Prostate + proximal 1cm of seminal vesicle	34 (81%)
Planning target volume (PTV) expansion from CTV	
3mm isotropic	12 (29%)
4mm isotropic	1 (2%)
4mm (except 3mm posterior)	3 (7%)
5mm isotropic	1 (2%)
5mm (except 3mm posterior)	23 (56%)
6mm (except 4mm posterior)	1 (2%)
Focal boost to intraprostatic lesions (n=42)	
No	23 (55%)
Yes	19 (45%)
Contouring of intraprostatic gross target volume (GTVboost) (n=19)	
MRI	19 (100%)
T2 weighted	18 (95%)
Diffusion weighted imaging (DWI)	11 (58%)
Dynamic contrast enhanced (DCE)	4 (21%)
PSMA PET	19 (100%)
Discordant between MRI and PSMA PET GTVboost (n=19)	
Boolean MRI and PSMA PET GTVboost	10 (53%)
Use MRI GTVboost	7 (37%)
Use PSMA PET GTVboost	1 (5%)
Omit boost GTV	1 (5%)
Planning target volume (PTV) for GTVboost (n=19)	
No	16 (84%)
Yes	3 (16%)
Urethra contouring	
CT urethrogram	4 (10%)
MRI	36 (86%)
Do you apply urethra planning organ at risk (PRV)	
0mm	6 (15%)
1mm	4 (10%)
2mm	18 (45%)
3mm	12 (30%)

*Abbreviations:* CT = computed tomography; CTV = clinical target volume; DCE = dynamic contrast enhanced; DWI = diffusion-weighted imaging; GTVboost = gross target volume; MRI = magnetic resonance imaging; PET = positron emission tomography; PTV = planning target volume; PSMA PET = prostate-specific membrane antigen positron emission tomography.

**Table-4:** Prostate SABR dose prescription, fractionation schedule and organs-at-risk dose constraints (n=41)

Prostate SABR treatment details	N (%)
Prescribed dose/ fraction to CTV	
36 Gy in 5 fractions	1 (2%)
36.25 Gy in 5 fractions	2 (5%)
40 Gy in 5 fractions	37 (90%)
42.7 Gy in 7 fractions	1 (2%)
Prescribed dose/ fractions to PTV	
36 Gy in 5 fractions	5 (12%)
36.25 Gy in 5 fractions	34 (83%)
40 Gy in 5 fractions	1 (2%)
42.7 Gy in 7 fractions	1 (2%)
Prescribed dose/ fractions to boostGTV (n=19)	
40 Gy in 5 fractions	5 (26%)
42 Gy in 5 fractions	5 (26%)
45 Gy in 5 fractions	8 (42%)
49 Gy in 7 fractions	1 (5%)
Fractionation schedule	
1 fraction/ week	8 (20%)
2 fractions/ week	21 (51%)
3 fractions/ week	32 (78%)
5 fractions/ week	2 (5%)
Which protocol’s dose constraints do you apply (select all that apply)	
PACE-B trial	20 (48%)
NINJA/ TROG18.01 trial	15 (36%)
eviQ*	17 (40%)

*Abbreviations:* CTV = clinical target volume; GTVboost = gross target volume; PTV = planning target volume.

⁎ eviQ = New South Wales Cancer Institute online evidence based treatment protocols.

### Treatment delivery

Most ROs (88%, 37/41) offer prostate SABR with a standard CT-based linear accelerator (LINAC), with four (10%) using CyberKnife and two (5%) using MR-LINAC (Table 5). Of the ROs treating patients with a standard CT-based LINAC, 84% (31/37) use intrafraction motion monitoring.

**Table-5:** Prostate SABR treatment delivery

Treatment delivery	N (%)
Treatment platforms (n=41)	
CT LINAC	37 (88%)
MR LINAC	2 (5%)
Cyberknife	4 (10%)
Intrafraction motion monitoring on CT LINAC (n=37)	
No	6 (16%)
Yes	31 (84%)
Truebeam triggered imaging	28 (76%)
Kilovoltage Intrafraction Monitoring (KIM)	3 (8%)
ExacTrac	3 (8%)

*Abbreviations:* CT = computed tomography; KIM = kilovoltage intrafraction monitoring; MR = magnetic resonance; LINAC = linear accelerator.

### Barriers

Twelve (23%) ROs currently do not offer prostate SABR. Barriers include a lack of clinical expertise and access to necessary technology (eg, intrafraction motion management, fiducial markers, and planning MRI), concerns about long-term outcomes, and competing departmental priorities (eg, brachytherapy) (Table 6).

**Table-6:** Barriers to prostate SABR implementation (n=12)

Barriers to prostate SABR implementation	N (%)
Lack of expertise	5 (42%)
Lack of intrafraction motion management	3 (25%)
Lack of access to fiducial marker	2 (17%)
Concern regarding long-term outcomes	2 (17%)
Lack of access to planning MRI	1 (8%)
Competing departmental priorities	1 (8%)

*Abbreviations:* MRI = magnetic resonance imaging.

## Discussion

This ANZ-wide survey provides a snapshot of contemporaneous prostate SABR patterns of practice, with important findings to guide the development of the binational FROGG prostate SABR guidelines.

Almost all ROs offer prostate SABR for intermediate-risk PCa, consistent with the patient group in the PACE-B trial.^1^ Approximately one-third offer prostate SABR for high-risk PCa, which may largely be driven by the currently recruiting NINJA trial^2^ in Australia. We anticipate the increasing use of prostate SABR for high-risk PCa with the expected opening of international trials such as NRG-GU013 (NCT05946213) and ASCENDE-SBRT (NCT06235697) in Australia. It is reassuring that no RO is currently offering prostate SABR for node-positive PCa off-trial^4^; this group of patients will be eligible to access prostate SABR in the NRG-GU013 trial.

Most ROs consider prostate volume as a patient selection criterion, given that large prostate volume may be associated with an increased risk of genitourinary adverse events. Most published trials did not have a prostate volume cutoff,^1^^,^^5^, ^6^, ^7^ while some currently recruiting or recently completed trials do, eg, 60 cm^3^ in ASCENDE-SBRT, 80 cm^3^ in SABRE, and 100 cm^3^ in NINJA^2^ and NRG-GU013. Baseline urinary function is also a key selection criterion. While most ROs assess baseline IPSS, only half reported using a specific cutoff (typically >20), similar to NINJA^2^ and ASCENDE-SBRT. There was no IPSS cutoff in the PACE-B trial, with 31% and 5% in the prostate SABR arm having moderate (IPSS 8-19) and severe (IPSS 20-35) symptoms at baseline, respectively.^1^ In the PACE-B post hoc analyses, a higher baseline IPSS score was associated with an increased risk of late grade >2 genitourinary adverse events,^8^ with an optimal suggested IPSS threshold of 11.

The majority of ROs use fiducial markers (93%) and rectal spacers (86%) for prostate SABR planning. While the PACE trials did not mandate fiducial markers, 73% and 44% of patients in the SABR arms of PACE-B and PACE-C, respectively, had fiducial markers. The benefit of rectal spacers for prostate SABR remains to be determined.^9^ Although a phase 3 randomized trial suggests that rectal spacers were associated with improved rectal dosimetry and reduced acute gastrointestinal adverse events, the benefit to bowel quality of life remains unknown.^10^ The currently recruiting SABRE trial may help answer this question.

Prostate MRI is becoming an integral component of prostate SABR planning, with nearly all ROs using MRI for prostate SABR contouring. In Australia, while diagnostic prostate MRI is currently funded under the Australian Medicare Benefits Schedule, prostate MRI for radiation therapy planning purposes is not funded, and the cost is covered by either patients or the treating department. Given the routine use of planning MRIs for prostate SABR, there may be a strong case to advocate for government-funded planning prostate MRIs.

The target volume contouring and dose fractionation used are generally consistent with published trial protocols, ie, 36.25 Gy in 5 fractions, delivered every other day.^1^ Some ROs offer weekly fractionation similar to the PATRIOT and NOVALIS trials.^11^^,^^12^ One RO offers 42.7 Gy in 7 fractions, similar to the HYPO-RT-PC trial.^13^^,^^14^ While HYPO-RT-PC is an ultrahypofractionated trial,^13^^,^^14^ it used non-SABR techniques, with most patients receiving 3-dimensional conformal radiation therapy (80%). Half of the ROs offer focal boosts for intraprostatic lesions in prostate SABR. While the FLAME trial^15^ showed oncological benefits of focal boosts in conventionally fractionated radiation therapy, data on focal boosts in prostate SABR are largely limited to single-arm phase 2 trials.^16^, ^17^, ^18^

Most ROs apply OAR dose constraints similar to those used in published or recruiting trials. While not specifically asked in the current survey, anecdotally most ROs tend to apply a stricter urethra dose constraint compared with that reported in the PACE trials, given that pooled analyses of >2000 patients treated with prostate SABR suggest that for every 1 Gy increase in urethra Dmax, there is a 1% increase in late grade >2 genitourinary adverse events.^19^

There are several limitations to this study. This was a voluntary survey, with a 28% response rate. Nonetheless, the response rate is not different from previous FROGG-commissioned surveys.^20^ Importantly, based on responders’ self-reported institutions, to our knowledge, we have captured responses from almost all institutions offering prostate SABR across all ANZ jurisdictions; thus, the survey is likely to reflect true ANZ-wide prostate SABR practice. However, survey nonresponders may include a higher proportion of ROs not currently offering prostate SABR. A qualitative study of current nonprostate SABR adopters may help to better identify barriers to adoption.

## Conclusions

In conclusion, this survey provides a snapshot of the contemporaneous prostate SABR practice in ANZ and insight into potential barriers to the adoption of prostate SABR. This information will be used to guide the development of a binational prostate SABR guideline to facilitate broader evidence-based adoption and implementation of prostate SABR for localized PCa across ANZ.

## Disclosures

Andrew Loblaw reports consulting fees from Bayer, Astellas, Knight, Tolmar, TerSera, and Sumitomo; honoraria from TerSera, AbbVie, Astellas, Bayer, Janssen, and Sanofi; travel support from Tolmar and TerSera; a patent on an endorectal immobilization device (GU-Lok); Chair of the Prostate Cure Foundation board; and his wife is the National Sales Manager, Oncology for Sanofi Canada. All other authors report no conflict of interest.

## References

[1] van As N., Griffin C., Tree A. (2024). Phase 3 trial of stereotactic body radiotherapy in localized prostate cancer. N Engl J Med.

[2] Martin J., Keall P., Siva S. (2019). TROG 18.01 phase III randomised clinical trial of the Novel Integration of New prostate radiation schedules with adJuvant Androgen deprivation: NINJA study protocol. BMJ Open.

[3] Ong W.L., Allan Hupman M., Davidson M. (2024). Urethra contouring on computed tomography urethrogram versus magnetic resonance imaging for stereotactic body radiotherapy in prostate cancer. Clin Transl Radiat Oncol.

[4] Ong W.L., Kang T.M.J., Loblaw A. (2025). Radiotherapy for node-positive prostate cancer in the PSMA-PET era: The need for prospective clinical trials. J Med Imaging Radiat Oncol.

[5] van As N., Yasar B., Griffin C. (2024). Radical prostatectomy versus stereotactic radiotherapy for clinically localised prostate cancer: Results of the PACE-A randomised trial. Eur Urol.

[6] Tree A.C., Hinder V., Chan A. (2025). Intensity-modulated moderately hypofractionated radiotherapy versus stereotactic body radiotherapy for prostate cancer (PACE-C): Early toxicity results from a randomised, open-label, phase 3, non-inferiority trial. Lancet Oncol.

[7] Kishan A.U., Ma T.M., Lamb J.M. (2023). Magnetic resonance imaging-guided vs computed tomography-guided stereotactic body radiotherapy for prostate cancer: The MIRAGE randomized clinical trial. JAMA Oncol.

[8] Ratnakumaran R., Brand D.H., Sasitharan A. (2026). Exploring factors associated with late urinary toxicity after prostate stereotactic body radiotherapy: Findings from the PACE-B study. Eur Urol.

[9] Harvey M., Ong W.L., Chao M. (2023). Comprehensive review of the use of hydrogel spacers prior to radiation therapy for prostate cancer. BJU Int.

[10] Mariados N.F., Orio P.F., Schiffman Z. (2023). Hyaluronic acid spacer for hypofractionated prostate radiation therapy: A randomized clinical trial. JAMA Oncol.

[11] Zilli T., Jorcano S., Bral S. (2023). Every-other-day versus once-a-week urethra-sparing prostate stereotactic body radiation therapy: 5-year results of a randomized phase 2 trial. Int J Radiat Oncol Biol Phys.

[12] Ong W.L., Quon H., Ong A. (2025). Every other day or once a week: Long-term oncological outcomes in the phase 2 PATRIOT trial of prostate stereotactic ablative body radiotherapy. Eur Urol Oncol.

[13] Widmark A., Gunnlaugsson A., Beckman L. (2019). Ultra-hypofractionated versus conventionally fractionated radiotherapy for prostate cancer: 5-year outcomes of the HYPO-RT-PC randomised, non-inferiority, phase 3 trial. Lancet.

[14] Nilsson P., Gunnlaugsson A., Beckman L. (2026). Ultra-hypofractionated versus conventionally fractionated radiotherapy for localised prostate cancer (HYPO-RT-PC): 10-year outcomes of an open-label, randomised, phase 3, non-inferiority trial. Lancet Oncol.

[15] Kerkmeijer L.G.W., Groen V.H., Pos F.J. (2021). Focal boost to the intraprostatic tumor in external beam radiotherapy for patients with localized prostate cancer: Results from the FLAME randomized phase III trial. J Clin Oncol.

[16] Draulans C., Haustermans K., Pos F.J. (2024). Stereotactic body radiotherapy with a focal boost to the intraprostatic tumor for intermediate and high risk prostate cancer: 5-year efficacy and toxicity in the hypo-FLAME trial. Radiother Oncol.

[17] Ramadan S., Loblaw A., Dhar A. (2025). PSMA MRI Guided prOstate SBRT (ARGOS)/Comprehensive, Longitudinal Evaluation of IMaging BiomarkErs Post Radiotherapy (CLIMBER) phase I/II trial. Int J Radiat Oncol Biol Phys.

[18] Ong W.L., Cheung P., Chung H. (2023). To boost or not to boost: Pooled analyses from 2-fraction SABR trials for localized prostate cancer. Int J Radiat Oncol Biol Phys.

[19] Leeman J.E., Chen Y.H., Catalano P. (2022). Radiation dose to the intraprostatic urethra correlates strongly with urinary toxicity after prostate stereotactic body radiation therapy: A combined analysis of 23 prospective clinical trials. Int J Radiat Oncol Biol Phys.

[20] Lawless A., Kneebone A., Armstrong S. (2023). Patterns of practice regarding use of radiation therapy following radical prostatectomy: A survey of Radiation Oncologists and Urologists in Australia and New Zealand. J Med Imaging Radiat Oncol.

